# Severe Perineal Lacerations in Obstetric Practice: The Effect of Institutional Practice Guidelines on Repair Failures in a Single Centre

**DOI:** 10.1155/2014/131682

**Published:** 2014-10-29

**Authors:** Shamir O. Cawich, Dwayne Wright, Santosh Kulkarni, Carol Rattray, Ian Bambury, Loxley Christie, Vijay Naraynsingh

**Affiliations:** ^1^Department of Clinical Surgical Sciences, University of the West Indies, St. Augustine Campus, St. Augustine, Trinidad and Tobago; ^2^Department of Obstetrics and Gynaecology, University of the West Indies, Mona Campus, Kingston, Jamaica

## Abstract

*Background.* There is a high incidence of failure after repair of severe perineal lacerations (SPLs). A tertiary referral hospital in the Caribbean introduced guidelines in an attempt to improve outcomes. We performed an audit of SPL repairs at this centre in an attempt to determine the effect on repair failure. *Methods.* All patients with SPL repairs between November 1, 2007, and December 30, 2012, were identified. The primary aim was to determine the incidence of failed repairs (wound dehiscence, anal sphincter disruption, rectovaginal fistula, and/or faecal incontinence). The Cleveland Clinic Incontinence Score (CCIS) was used to assess continence at discharge and 24 weeks after repair. Data were analyzed with SPSS version 12. *Results.* There were 8108 vaginal deliveries, 23 third-degree injuries, and 3 fourth-degree injuries. Three patients experienced a repair failure. Notably, 69% of surgeons chose an inappropriate suture for sphincter repair. *Conclusions.* Experienced operators are performing repairs, but there is a high prevalence of inappropriate suture choice for repairs. A targeted educational campaign may be necessary to remind clinicians of the best practice in repair techniques.

## 1. Introduction

Women develop severe perineal lacerations (SPLs) involving the anal sphincters during 0.5% [1–3] to 6% [4] of vaginal deliveries. In these circumstances, an urgent perineal repair is required. This involves several maneuvers, including dissection and reconstruction of the anal sphincter complex and anatomic reapproximation of perineal tissues. Normal anorectal function depends heavily on the integrity of the perineal repair. When the repair fails, a rectovaginal fistula results from perineal wound dehiscence and sphincter disruption leads to faecal incontinence.

We performed an audit at a single centre in Jamaica evaluating patients who had SPL repairs between 2004 and 2006 [5]. This revealed that 0.2% of women sustained SPLs [5], which was lower than expected when compared to international figures [2–4]. However, there was a high incidence of failed repairs, with 29% of women developing fistulae and/or incontinence [5]. Fortuitously, the audit identified three pitfalls in SPL repair that could be changed: inexperienced operators, inappropriate suture choice, and inappropriate repair techniques [5]. These were addressed through continuing education for clinicians in obstetric practice and policy change mandating repair by experienced staff and the development of institutional guidelines for SPL repair [6–9]. These measures were instituted in 2007 in an attempt to reduce failure rates [6]. The current study sought to evaluate the effect, if any, that these measures had on failure rates at a single centre in Jamaica.

## 2. Method

The institutional review board granted permission to carry out an audit at the University Hospital of the West Indies, one of two referral hospitals serving a catchment population of 826,880 persons in and around the nation's capital, Kingston [11].

We accessed the labour ward records to identify all consecutive patients who had vaginal deliveries between November 1, 2007, and December 30, 2012. All patients who sustained SPLs were identified and their records were reviewed. The following data were extracted: sphincter injury details, suture choice, repair technique, and details of surgeons performing the repair.

The primary aim was to document the incidence of failed repairs. A failed repair was considered present when there was wound dehiscence, sphincter disruption, rectovaginal fistula, and/or faecal incontinence. Perineal examination was performed to detect wound dehiscence that was considered present when the deep layers of the sutured perineum became separated. Sphincter disruption was considered present when the sphincter edges were not apposed on physical examination. A rectovaginal fistula was considered present when there was a communication between the epithelial surfaces of the vagina and the anorectum. The Cleveland Clinic Incontinence Score (CCIS) was used to evaluate continence because it was practical and easy to use and considered lifestyle alterations (Table 1). Faecal incontinence was defined as the inability to retain stool or gas and expel it at a proper time and place and was considered present with a CCIS > 7 [10]. The patients were routinely assessed at time of hospital discharge and 24 weeks after repair. The CCIS was calculated at each assessment to evaluate continence.

A secondary aim of this study was to detect any change in the three management pitfalls previously identified (improper suture choice, poor repair technique, and inexperienced operators) after institutional management guidelines for SPL repair were introduced in 2007 [5]. Slowly absorbable or nonabsorbable sutures were considered appropriate for sphincter repair. We considered attending grade surgeons/obstetricians and postgraduate residents in their final year of training as experienced operators.

The institutional management guidelines called for anatomically correct repair of SPLs in the following layers: rapidly absorbable sutures to repair vaginal mucosa, slowly absorbable sutures to repair perineal musculature/perineal body, complete mobilization of the anal sphincters, sphincter approximation with slowly absorbable or nonabsorbable sutures using either the overlapping or end-to-end technique, and rectal mucosal repair with rapidly absorbable sutures. Due to the retrospective nature of the study, however, we could not find a way to objectively assess repair technique since we could only evaluate the surgeons' records and their description of the technique. Therefore, the technique used was recorded for descriptive purposes and both end-to-end and overlapping techniques for sphincter repair were considered appropriate.

The Statistical Package for Social Sciences version 12 software (SPSS Inc., Chicago, IL, USA) was used for data management and statistical analysis on data sets. We compared the outcomes between this study period and the raw data collected in the previous audit (phase 1). Descriptive analyses (cross tabulations, frequencies, and descriptive ratio statistics) were generated using the populated data spreadsheets. Chi-squared tests and Fisher's exact tests were used to assess associations. Student's* t*-test was used to compare means between variables of interest.

## 3. Results

There were 8,108 vaginal deliveries over the study period. Severe perineal lacerations occurred in 26 women (0.32%) at a mean age of 27 ± 5.78 years (range 17–38). There were 23 third-degree injuries and 3 fourth-degree injuries. The incidence of SPLs had not changed significantly compared to phase 1 in which there were 8 SPLs from 3957 consecutive vaginal deliveries (0.2%).

Experienced clinicians performed all the repairs in this series: attending grade clinicians (11) and residents in their final year of postgraduate training (15). The anal sphincter complex was reconstructed with appropriate sutures in 8 cases (31%) and with rapidly absorbing polyglactin (inappropriate) sutures in the remaining 18 (69%).

The operative technique for sphincter repair was not clearly described in 5 cases (19%). In the remaining cases, an overlapping sphincter repair technique was used in 8 (31%) and end-to-end technique in 13 (50%). To complete the repair, 2/0 or 3/0 vicryl sutures were used to repair the rectal mucosa in 24 (92%) cases. No stomas were constructed in this series.

In this series, three patients experienced a failure of the perineal repair (12%). The failure rates in multiparous women (1/11) and primiparous women (2/15) in this study period were similar (9.1% versus 13.3%; *P* = 0.34). There was a trend toward a reduced incidence of repair failures compared to that in phase 1 (24% versus 12%; *P* = 0.282).  Table 2 documents the primary and secondary outcomes of this study and compares them with the raw data from phase 1 study.

## 4. Discussion

In an attempt to reduce perineal repair failure rates, institutional guidelines and policy changes were implemented at this facility in 2007 [6]. This resulted in a downward trend in the incidence of repair failures (29% versus 12%), but it did not achieve statistical significance. We recognize that the small study population makes statistical analysis weak, but SPLs are uncommon in Caribbean obstetric practice [5, 7]. It would take several decades to accrue a large case series.

In this series, experienced operators performed all the perineal repairs. This was significant improvement over phase 1 where 43% of the operators were junior level staff. This might have contributed to the downward trend in failure rates during phase 2.

However, when we analyzed suture choice, we found that 69% of surgeons chose a rapidly absorbable suture to reconstruct the sphincter. These sutures are inappropriate for sphincter repair because they are absorbed before the muscle has had time to heal [12–17]. Nonabsorbable or slowly absorbing sutures are recommended as a better choice for sphincter reconstruction [12, 13]. Although there was some improvement over phase 1, when 86% of operators chose an inappropriate suture, it remained unacceptable for 69% of sphincters to be reconstructed with rapidly absorbable sutures in phase 2.

There was an increase in the utility of the overlapping sphincter repair technique in phase 2 (31% versus 14%). Although the end-to-end and overlapping repairs were both recognized to be reasonable options for sphincter repair, the overlap technique is being used increasingly because it theoretically results in more tissue contact after repair and better long term healing [12, 13, 16, 18]. We considered this a positive trend in phase 2. However, in an era when much effort was put into correcting pitfalls in SPL repair, it was disappointing that the operative notes had an unclear description of the sphincter repair technique in 19% of cases. This may reflect a lack of appreciation of the importance of repair technique, disregard for the best practice recommendations, or ineffective implementation of institutional practice guidelines. Due to the nature of the study, we were not able to analyze this in depth, but this is an important aspect for future studies to analyze.

Despite the continued use of inappropriate sutures in 69% of cases, there was a trend toward reduced failure rates (29% versus 12%) in phase 2. It is possible that the increase in experienced operators may have offset the suture choice to account for this change. We can only surmise that there may have been a further reduction in failure rates if appropriate sutures were utilized.

The study mechanism did not allow us to evaluate the other factors that have been touted as contributors to failure such as the underdiagnosis, underestimation of injury severity, and poor understanding of perineal anatomy [12, 19]. The continued education for clinicians in obstetric practice was intended to address these, but we were unable to assess their contribution due to the nature of this study.

## 5. Conclusion

The incidence of SPL has remained unchanged at this facility. There has been a trend toward reduced failure rates after perineal repair, although it has not attained statistical significance. Experienced operators are performing significantly more repairs, but a targeted educational campaign may be necessary to remind these operators of the best practice in repair technique.

## Figures and Tables

**Table 1:** Cleveland Clinic Incontinence Score.

	Gas	Liquid stool	Solid stool	Use of pads
Occasionally	1	4	7	1
>1 per week	2	5	8	2
Daily	3	6	9	3

Cleveland Clinic Incontinence Index (IC):				
CCIS	0	Perfect continence		
CCIS	1–7	Good continence		
CCIS	8–14	Moderate incontinence		
CCIS	15–20	Severe incontinence		
CCIS	>20	Complete incontinence		

Reproduced with permission from [10].

**Table 2:** Comparison of therapeutic outcomes in SPL repair.

Parameter evaluated	Phase 1 (*n* = 7)	Phase 2 (*n* = 26)	*P* value
Details of perineal repair			
Experienced staff performed repair	4 (57%)	26 (100%)	**0.01**
Appropriate suture for sphincter repair	1 (14%)	8 (31%)	0.64
End-to-end sphincter reconstruction	6 (86%)	13 (50%)	0.20
Overlapping sphincter reconstruction	1 (14%)	8 (31%)	0.64
Therapeutic outcomes			
Failure of perineal repair	2 (29%)	3 (12%)	0.282
Wound dehiscence/rectovaginal fistula	1	2	0.524
Sphincter disruption/faecal incontinence	2	3	0.282

## References

[1] Sultan A. H. (1999). Perineal injury and fecal incontinence after childbirth: obstetrical perineal injury and anal incontinence. *Clinical Risk*.

[2] Sultan A. H., Kamm M. A., Hudson C. N., Bartram C. I. (1994). Third degree obstetric and sphincter tears: risk factors and outcome of primary repair. *British Medical Journal*.

[3] Tetzschner T., Sorensen M., Lose G., Christiansen J. (1996). Anal and urinary incontinence in women with obstetric anal sphincter rupture. *British Journal of Obstetrics and Gynaecology*.

[4] Carroll T. G., Engelken M., Mosier M. C., Nazir N. (2003). Epidural analgesia and severe perineal laceration in a community-based obstetric practice. *Journal of the American Board of Family Practice*.

[5] Cawich S. O., Mitchell D. I. G., Martin A. (2008). Management of obstetric anal sphincter injuries at the University Hospital of the West Indies. *West Indian Medical Journal*.

[6] Cawich S. O., Mitchell D. I. G., Martin A. (2007). Can we improve therapeutic outcomes after obstetric anal sphincter injury at the University Hospital in Jamaica?. *West Indian Medical Journal*.

[7] Lewis T., DaCosta V., Harriott J., Wynter S., Christie L., Cawich S. (2011). Factors related to obstetric third and fourth degree perineal lacerations in a Jamaican cohort. *The West Indian Medical Journal*.

[8] Cawich S. O., Bambury I., Mitchell D. I. G., Plummer J., Newnham M. S., Christie L. (2008). Is a diverting colostomy required after repair of obstetric anorectal injuries?. *The Internet Journal of Third World Medicine*.

[9] Cawich S. O., Bambury I., Mitchell D. I. G., Plummer J. M., Williams E. W. (2007). Colostomy for a fourth degree perineal laceration: where is the evidence?. *International Journal of Gynecology & Obstetrics*.

[10] Altomare D. F., Baeten C. G. M. I., Bazzocchi G. Consensus conference on treatment options for fecal incontinence. http://www.colorep.it/rivista%20CEC/consensus_conference.htm.

[11] Cawich S. O., Harding H. E., Crandon I. W. (2013). Leadership in surgery for public sector hospitals in Jamaica: strategies in the operating room. *The Permanente Journal*.

[12] Fernando R. J., Sultan A. H., Radley S., Jones P. W., Johanson R. B. (2002). Management of obstetric anal sphincter injury: a systematic review & national practice survey. *BMC Health Services Research*.

[13] Leeman L., Spearman M., Rogers R. (2003). Repair of obstetric perineal lacerations. *The American Family Physician*.

[14] Adams E. J., Fernando R. J. (2001). RCOG green top guidelines: management of third and fourth degree perineal tears following vaginal delivery. *Green Top Guidelines*.

[15] Mahomed K., Grant A., Ashurst H., James D. (1989). The Southmead perineal suture study. A randomized comparison of suture materials and suturing techniques for repair of perineal trauma. *British Journal of Obstetrics and Gynaecology*.

[16] Parks A. G., McPartlin J. F. (1971). Late repair of injuries of the anal sphincter. *Proceedings of the Royal Society of Medicine*.

[17] Grant A. (1989). The choice of suture materials and techniques for repair of perineal trauma: an overview of the evidence from controlled trials. *British Journal of Obstetrics and Gynaecology*.

[18] Sultan A. H., Monga A. K., Kumar D., Stanton S. L. (1999). Primary repair of obstetric anal sphincter rupture using the overlap technique. *British Journal of Obstetrics and Gynaecology*.

[19] Sultan A. H., Kamm M. A., Hudson C. N. (1995). Obstetric perineal trauma: an audit of training. *Journal of Obstetrics and Gynaecology*.

